# A review of burnout in college English teachers in China

**DOI:** 10.3389/fpsyg.2022.884304

**Published:** 2022-08-02

**Authors:** Yurong Zheng

**Affiliations:** Foreign Language College, Changchun University, Changchun, China

**Keywords:** college English teacher, teacher burnout, burnout causes, teacher support, burnout alleviation

## Abstract

College English teachers’ job burnout has become prominent in the field of education. Using China National Knowledge Infrastructure (CNKI) database, this review research on burnout of college English language teachers in China from 2006 to 2021. The review demonstrates key research areas including teacher burnout severity and influencing variables related to teacher burnout. Individual factors, such as age, gender, marital status, educational background, professional title, and years of teaching experience are associated with burnout rates. University type and level, teaching-related role overload, scientific research stress induced by promotion, limited job autonomy, a stern hierarchical organizational system, and opaque operating rules are influencing factors discussed. Possible ways to reduce burnout across micro, meso, and macro-levels, along with practical implications and limitations are discussed.

## Introduction

Burnout is a prolonged response to chronic emotional and interpersonal stressors experienced at work and is characterized by the three dimensions: exhaustion, cynicism, and inefficacy (Maslach et al., 2001). Since the 1980s, teaching has been considered a high-risk profession due to the high number of risk factors that impact teacher health and wellbeing. As such, burnout in educators has drawn attention from researchers. Teachers are required to respond to a wide range of stressors and resultantly tend to report high levels of occupational stress and burnout (Garrick et al., 2014). Teachers experience burnout owing to depletion of emotional resources, depersonalization (DP) of students, and a lack of personal accomplishment, which has a substantial impact on teachers’ professional functioning (Maslach, 2003; Klusmann et al., 2008).

In recent years, the job burnout of teachers is drawing the attention of more and more researchers in China. Using the keyword “teacher career burnout,” a total of 2,741 research papers within the time span of 2000–2021 can be retrieved from the search engine of the China National Knowledge Infrastructure (CNKI) database. Literature review show that Chinese domestic studies in teacher burnout field began rather late and remain at a low level still. most empirical studies of the job burnout of teachers are descriptive, and the scales employed in the studies are directly borrowed from abroad (Tang, 2020). Factors contributing to the burnout of teachers are normally deduced in the way of empirical generalization and rational speculation. In addition, some problems can be found in relation to the correct use of those scales and the final measurement results leading to some conclusions lack in reliability and comparability (Jiang, 2018).

Economic globalization has resulted in language communication becoming increasingly important. The Chinese government has emphasized the importance of teaching English, which has resulted in the continuous higher educational reform and development. Therefore, college teachers’ jobs have come under increasingly high pressure (Yin, 2011). This literature review aims to synthesize Chinese studies on college English teachers’ burnout, not other English teachers. College English is a special term referring to the general English courses for 41.83 million students enrolled at higher education level on records (2021) since students’ compulsory Foreign Language credits are overwhelmingly finished choosing English. Though there is no evidence that English teachers experience higher level of burnout than teachers teaching other subjects in China since there is no such comparative research found from CNKI database. One round of topic search result from CNKI database using the keyword “teacher career burnout” coupled with “college teacher” shows a total of 128 papers in Chinese retrieved. 73 papers (57%) among which match the keyword “teacher career burnout” and 61 papers (48%) carry the keyword “College English teacher” in major topic category. This preliminary data screening finds that as a considerable part of studies on teacher burnout, most of the papers published in Chinese identify research trends regarding Chinese English teacher’s level of burnout as a major focus. Job burnout in college English teachers is a prominent area of enquiry in the field of education and has been extensively researched around the world (Tang, 2011). College English teacher burnout is an issue that continues to attract Chinese researchers’ attention. Chinese researchers have studied the effect of job burnout on college English teachers, drawing on 30 years of Western scholarship (Hu, 2016). This literature review aims to synthesize Chinese studies on college English teachers’ burnout and identify research trends and indicate limitations to provide insights regarding certain directions for future investigation. This review further aims to identify strategies to alleviate the pressure on college English teachers and identify countermeasures to better English as a Foreign Language (EFL) classroom efficiency and promote college English teachers’ career development in China.

## Research design

### Research question

This review examined literature on the burnout of English language teachers in China from 2006 to 2021 within the CNKI database. This review aimed to explore (a) the overall level of burnout in college English teachers; (b) the internal and external variables related to the burnout of college English teachers; and (c) burnout countermeasures in the classroom.

### Literature search

China Academic Journals full-text database (CNKI) database was used for research on burnout in college English Teachers in China from 2006 to 2021. All articles searched were published in Chinese. The search term: “teacher (or educator) burnout” was used for the first-round selection using CNKI search engine. The search resulted in 3,048 unique entries. As our target subjects are teachers teaching English for general purposes (EGP) in universities and colleges, which are called college English, a second cross search using the search term “college English” narrowed the articles down to 96 unique entries. The search terms expanded to include: “university English teacher,” “college English teacher,” “(EGP),” and “job burnout” to further cross search using the search engine to avoid omission. The titles, abstracts, and keywords of the 96 articles were manually examined. A final sample of 68 articles were selected for this systemic review.

## Results and discussion

### Description of scientific research into teacher burnout in China

College Teacher burnout has been increasingly researched over the last 15 years. From 2006 to 2011, only 15 articles were published. Only one master’s thesis was published during this period (Luo, 2007). During the following years, 10 other masters and doctoral degree theses were written on the same topic. Comparatively, from 2012 to 2017, 24 articles were published. From 2017 to 2021, 29 articles were published, with 9 featuring in the Peking University core periodical catalog.

**FIGURE 1:**
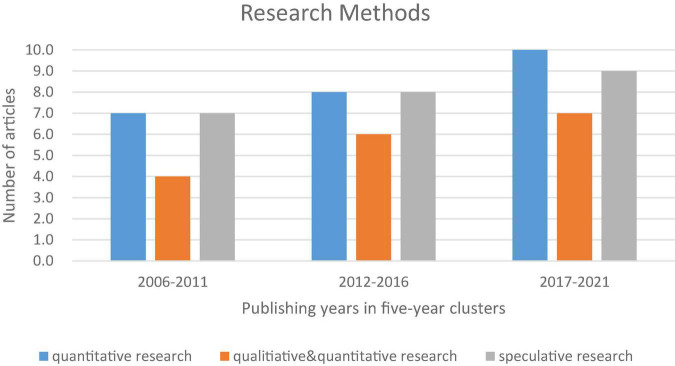
Research methods for selected journal articles.

Teacher burnout has predominantly been studied using empirical studies, both globally and in China. Of the 68 articles identified for this review, 42 articles (62%) used an empirical research design. Twenty-five of 42 articles used quantitative approaches and 17 articles used both quantitative and qualitative approaches, as shown in Figure 1.

Tang et al. (2018) stated that additional research, using both quantitative and qualitative research methodologies, should be conducted to examine teacher burnout in China.

### Key research areas on teacher burnout in China

We identified three main research areas in the selected articles to identify key research areas: teacher burnout severity, influencing variables and factors related to teacher burnout, and countermeasures and strategies to address teacher burnout.

#### Teacher burnout severity

Burnout is characterized by three symptoms (Maslach, 1986): (1) Emotional Exhaustion (EE), which is defined as the feeling of being physically and emotionally overextended; (2) DP, which is defined as a distant attitude toward students; and (3) a loss of self-confidence and lack of personal accomplishment (RPA). Influential Western scholarship studied the severity of teacher burnout through self-report measures constructed around the above mentioned three dimensions such as Maslach Burnout Inventory (MBI-Educators-Survey, MBI-ES) (MBI; Maslach et al., 1996). Using the same assessment tool, some Chinese researchers conducted their survey to measure the presence and severity of teacher burnout (Cheng, 2005; Wu, 2010). Some researchers revised the MBI-ES to suit the Chinese context. For example, Guohong and Linan (2008) developed a measure to assess a physical educator’s stress level. In synthesizing Chinese studies, different tools used are considered. This review tries to balance this situation by selecting even number of research using MBI-ES standards and revised MBI-ES, and the self-composed inventories are included as well to conclude the general trend as a finding.

Table 1 shows the main results from 10 papers assessing the level of Chinese college teacher burnout.

**TABLE 1:** Main results of burnout from 10 studies.

Researchers	Subjects targeted	Tools used	The status or the severity of burnout
Cheng (2005)	290 Chinese EFL teachers	MBI-ES	Burnout syndrome is not serious of the subjects indicated by EE, DP, and RPA value results.
Guo and Wang (2018)	65 college English teachers	Modified college working stress scale and MBI-ES	Stress and burnout level are in the middle of the scale
Tang (2011)	106 college EFL teachers	MBI	The comprehensive level of burnout is moderate in 46% and severe in 8% subjects.
Zhang (2012)	300 college English teachers	Revised MBI	The level of burnout is severe in subjects
Su (2014)	53 EFL teachers of post-80s generation	MBI-ES	The degree of college English teachers of post-80s generation is low but younger teachers are more likely to have job burnout.
Hu (2016)	123 college English teachers in Fujian	Revised MBI-ES	57% of college English teachers had serious EE.
Fang (2017)	143 EGP teachers	Modified MBI-ES	The job burnout of female teachers with lecturer title is obvious.
Xu (2010)	240 participants	Chinese MBI-ES and a socio-demographic; inventory	(75.4%) Had scores indicative of job burnout.
Chen and Lin (2008)	150 college English teachers in Guangzhou	Self- composed MBI	Overall burnout is obvious with severity in EE and RPA.
Liu (2009)	180 college English teachers	MBI	High in EE and RPA but low in DP.

The results of these studies demonstrate that Chinese college English teachers have a moderate and mild state of job burnout, whereas some research shows high burnout level (Tang, 2011). A few studies expand the definition theory of burnout beyond the three dimensions defined above. Gu (2016) tried in terms of theoretical perspective. He elaborates ecological dynamic system theory in teacher emotion research and demonstrates the explanatory power of this theory with his empirical study. He conceptualizes Chinese university EFL teachers’ emotion framework and offers a case-based theoretical framework to understand teacher emotions in the Chinese context. Liu (2019) integrates the identity theories from the sociocultural and psychological perspective to make a comprehensive analysis of the university EFL teachers’ professional identity development. By constructing a theoretical model of teacher’s professional identity development, he enriches the theoretical perspectives on college teacher burnout.

A study conducted by Xu (2010) in six universities indicates that of the 240 participants, 181 (75.4%) had scores indicative of job burnout. Of the participants who indicated burnout, 109 (45.4%) scored a low level of burnout, 59 (24.6%) were at a moderate burnout level, and 13 (5.4%) reported a high burnout level. Furthermore, 60 (25.0%) participants had a high score for EE, 81 (33.8%) had a high score for DP, and 125 (52.1%) had a high score for RPA. Hu (2016) demonstrated that there is a certain degree of job burnout among college English teachers in Fujian ordinary universities. Among the three dimensions, 57% of college English teachers had serious EE, but DP and RPA were non-severe.

#### Influencing variables and factor related to teacher burnout

There is no consensus on the causal factors related to educator burnout in China. Most of the articles explore internal or external factors related to teacher burnout. Factors related to teacher burnout are categorized into micro- (individual factors), meso- (organizational factors), and macro- (social factors) dimensions.

Micro-dimensions analyze how teachers’ individual differences impact teacher burnout. Many studies have been conducted on the relationship between burnout and individual factors, such as age, gender, and marital status (Mousavy and Nnmehchisalem, 2014). The selected articles demonstrate that educational background, professional title, and years of teaching experience are associated with burnout rates. The degree of burnout does differ significantly by gender (Xie, 2013; Liang, 2016). Liang (2016) reached the conclusion that the teachers aged below 30 scored the highest in EE, and PA is associated with older teachers and those with higher education. Xie (2013) demonstrated that years in teaching was not significantly related to dimensions of EE and DP but noted significant differences in PA. English teachers with 1–10 and 11–20 years of teaching experience had similar PA, but both had significantly higher PA than those with more than 20 years teaching experience. Most studies do not consider educational background as having a significant effect on job burnout in college English teachers. However, few researchers stated that teachers with a master’s degree experienced the highest degree of job burnout along all three dimensions and had a higher overall level burnout (Liu L., 2014). No significant correlation was found between teachers’ professional title and college English teacher burnout (Xu, 2010; Xie, 2013; Liu L., 2014). Zhang (2014) revealed that teachers with a junior title report the most serious burnout compared to those with a senior title, who experience a comparatively lower level of burnout.

Self-efficacy is the term that worth being singled out that is closely related with occupational self-concept in teacher burnout. According to the social cognitive theory, teacher self-efficacy is considered to have close and intrinsic link with both occupational stress and teacher burnout. Liu P. (2014) states that there is a negative correlation between college English teachers’ self-efficacy and job burnout. The improvement of college English teachers’ self-efficacy is conducive to reducing occupational burnout (Zhou, 2016). Teacher self-efficacy, occupational stress and teacher burnout are significantly correlated with each other. Han (2013) indicates that young female college English teachers experience high level of self-efficacy and occupational stress, but low level of burnout and moderately high level of EE. Another research shows that male teachers are better equipped with self-efficacy than that of women, and the degree of job burnout of women is higher than that of men; the self-efficacy of college English teachers with professional titles is better than that of college English teachers without professional titles (Wu and Wang, 2018). Domestic research also shows that teachers’ self-efficacy is the cause of job burnout (Liu, 2004). In addition, if the teacher’s self-efficacy is too low, it is easy to leave teaching career due to occupation burnout (Hu and Jiang, 2006).

Seminal Western research considers the meso-dimension, such as organizational factors (Carroll and White, 1982). All teachers interact with students within an organization framework. In fact, many researchers accept that teachers’ individual characteristics as well as job-related stressors should be considered when studying burnout (Kokkinos, 2007). High levels of burnout in higher vocational teachers indicates that university type and level are important variables for the study of burnout (Tang et al., 2018). Possible reasons for Chinese teachers’ turnover rates include a high level of stress, student behavior, and heavy workload (Liu and Onwuegbuzie, 2012). Work overload and rapid economic growth results in the deterioration of teachers’ mental health in China (Yang et al., 2019). Some researchers confirm that over-loaded work stress may lead to college English teacher burnout and what it involved such as self-development and contradiction between examination and innovation affect some of dimensions of burnout (Tang, 2011; Wei, 2011). Wei (2011) pointed out that student’s behaviors, and the scarcity of teachers’ career development or self-development opportunities also influence teachers’ EE and further effect college English teacher burnout (Zhang, 2014). In terms of teacher professional development, Gong and He (2018) outline 10 environmental burnout elements, including overload, severity of students’ problems, lacking supervisors’ support, limited job autonomy, a stern hierarchical organizational system, and opaque operating rules.

Macro-dimensions of teacher burnout indicate that social expectations and pressure on college English teaching effect teacher burnout (Tang, 2011). Some studies have shown that teaching-research conflict was positively associated with EE and DP (Heng et al., 2020). Tang (2011) extends this research and states that teaching-research conflict and school regulations are positively associated with the level of teacher burnout. The low salary of college English teachers and associated low socioeconomic status may increase teachers’ psychological risks (Liu and Onwuegbuzie, 2012). Due to heavy workload and short working hours, low professional titles, low wages, and limited social resources, college English teachers face pressure to support their families. When faced with the “survival” and “development” contradiction, people must ensure their survival. Only by solving the problem of survival, can development be a possibility. When teachers in colleges and universities must make this choice, survival will occupy the space of development. College English teachers must focus on their livelihood. Their energy in teaching and scientific research will naturally decrease and their professional development will slow down. Therefore, college English teachers’ survival in society is difficult, as they face a multitude of challenges.

#### Countermeasures and strategies

The selected studies mostly suggested countermeasures drawing on the perspectives of teachers themselves. From a teacher’s personal and professional development perspective, Hu (2016) found that improving self-efficacy, developing reflective teaching, and using peer-assisted learning are strategies to reduce teacher burnout. He argues that self-efficacy could function as a mediating variable that would reduce the negative effects of contextual variables and stressors. Many researchers proposed enhancing pressure resistance through internal and external resources (Liu P., 2014; Liang, 2016). These studies emphasized combining psychological capital, as an internal resource, with external resources to accomplish the harmonious unity of the two. Liang (2016) states that maintaining a positive attitude, redefining oneself, and career plan development alleviate pressure on teachers. Qualitative studies (Liu L., 2014) provide additional suggestions, including cultivating the sense of belonging, consolidating teachers’ identity, strengthening a sense of responsibility and enhancing teachers’ emotional labor management strategies.

Scholarship has also examined external strategies to address teacher burnout. Tang (2020) demonstrates that teachers’ professional identity, to certain degree, plays an intermediary role in teachers’ professional learning community and teachers’ job burnout, indicating that teachers’ professional learning community not only enables direct and forward prediction of teachers’ job burnout, but also imposes indirect influence on teachers’ job burnout *via* teachers’ professional identity. This intervention is in line with the research conclusions done by Lee et al. (2011) and Zonoubi et al. (2017). Which prove that professional learning community can improve teachers’ teaching efficacy and reduce burnout through professional practice and application together.

Wang (2007) suggests a scientific management system and humane mechanism to address the root cause of teacher burnout. Universities should reform teacher evaluation system to form a compound evaluation system which combines formative evaluation and final evaluation instead of the relying on the current professional title evaluation centered incentive feedback mechanism. Wang (2007) proposes that universities should implement an open democratic management style and provide teachers greater professional autonomy and freedom. This will assist teachers maintain their enthusiasm and reach their full potential. Excessively centralized control in teaching (Yang et al., 2019), limited job autonomy, a stern hierarchical organizational system, and opaque operating rules (Gong and He, 2018) are influencing factors for teacher burnout. Thus, switching to an academically oriented school administration may enhance resilience and wellbeing among teachers. Gong and He (2018) and Yang et al. (2019) indicate those suggestions are differ in the specific context of college English teachers in China than worldwide.

## Implications and conclusion

Our systematic review of research from 2006 to 2021 has demonstrated an increase of research on burnout in college English teachers. The qualities of the literature in this review are trusted sources in Chinese research database as they are mostly published in journals that ranked on Peking University core periodical catalog. Despite teacher burnout receiving global attention, Chinese specific situations and demands are noted. Liu P. (2014) pointed out that college foreign language teachers’ job burnout can also cause exhaustion of knowledge. Burnout teachers feel boring and lost desire for knowledge in their subject area, and their professional life is in a state of exhaustion. Most intervention and counter measures suggested by Chinese researchers are in line with studies done outside China to prove that appropriate intervention projects at the individual and organizational levels for job burnout has a positive effect on job burnout and mental health of practitioners (Taormina and Law, 2000; Zanni, 2008). There is no lack of its unique feature in specific context of China though. One aspect of burnout causes that set college English teacher burnout in China apart from the international literature is the evaluation systems which set the same requirement for all teachers irrespective of subjects they teach. Most college English teachers undertake heavy teaching tasks, and there exists dual pressure of teaching and scientific research. They are in the era facing elimination of publish or perish (Fan and Yang, 2015). The number of journals in linguistics are so small within China that it causes high pressure for the huge number of college English teachers in China to publish papers to pass their university’s research evaluation. Unreasonable school management and evaluation systems and lack of resources and opportunities put teachers at an overload working state, physical and mental exhaustion (Sun, 2011).

There are several limitations in the current body of scholarship on college English teacher burnout. First, research subject matter is limited. Studies that focus on factors other than burnout level, influencing variables, and burnout countermeasures and strategies are rare. Second, the quality of the research is low. Many studies in the field are speculative; thus, more in-depth research is needed. Third, most of the research is practical and few attempts made on theoretical expansion based on China’s context. No experimental research was conducted to measure the effectiveness of the countermeasures suggested in the selected studies.

Teacher burnout is an important phenomenon that affects the education system and society. Additional research is needed for the study of teacher burnout in China. Both qualitative, quantitative, and theoretical research would assist in refining the study of college English teacher burnout. Additional research should be conducted on external control and internal drive. Research should be conducted at both regional and national level. In addition, longitudinal studies or studies focusing on the effectiveness of burnout interventions should be conducted (Jiang, 2018).

The current review has three major implications. Scholarship on job burnout in college English teachers should be reviewed by related government departments, the teaching guidance committee, and the teaching research association. College administration should examine the job burnout of college English teachers from the perspective of higher education management. College English teachers are an important group of teachers in colleges and universities. Despite this, they have not received the required attention of university management due to the nature of public basic courses. University administration should actively assess college English teachers’ job burnout to understand current levels of burnout and the main influencing factors. Furthermore, college English teachers themselves need to have a clearer understanding of job burnout. More attention should be paid by the university management to the three dimensions of job burnout, which lays the foundation for effective intervention or prevention of college English teacher burnout. Further studies are warranted to establish how various countermeasure strategies impact teachers’ effective teaching in EFL language classrooms.

## Author contributions

The author confirms being the sole contributor of this work and has approved it for publication.
